# Traumatic Brain Injury Activation of the Adult Subventricular Zone Neurogenic Niche

**DOI:** 10.3389/fnins.2016.00332

**Published:** 2016-08-02

**Authors:** Eun Hyuk Chang, Istvan Adorjan, Mayara V. Mundim, Bin Sun, Maria L. V. Dizon, Francis G. Szele

**Affiliations:** ^1^Samsung Biomedical Research Institute, Samsung Advanced Institute of Technology, Samsung Electronics Co., Ltd.Seoul, South Korea; ^2^Department of Physiology, Anatomy and Genetics, University of OxfordOxford, UK; ^3^Department of Anatomy, Histology and Embryology, Semmelweis UniversityBudapest, Hungary; ^4^Department of Biochemistry, Universidade Federal de São PauloSão Paulo, Brazil; ^5^Department of Pediatrics, Prentice Women's Hospital, Northwestern University Feinberg School of MedicineChicago, IL, USA

**Keywords:** adult neurogenesis, stem cells, traumatic brain injury (TBI), regeneration, proliferation

## Abstract

Traumatic brain injury (TBI) is common in both civilian and military life, placing a large burden on survivors and society. However, with the recognition of neural stem cells in adult mammals, including humans, came the possibility to harness these cells for repair of damaged brain, whereas previously this was thought to be impossible. In this review, we focus on the rodent adult subventricular zone (SVZ), an important neurogenic niche within the mature brain in which neural stem cells continue to reside. We review how the SVZ is perturbed following various animal TBI models with regards to cell proliferation, emigration, survival, and differentiation, and we review specific molecules involved in these processes. Together, this information suggests next steps in attempting to translate knowledge from TBI animal models into human therapies for TBI.

## Introduction

Traumatic brain injury (TBI) is a common problem with approximately 53,000 civilians dying from it annually in the United States alone and with as many as 5.3 million people living with TBI-related disabilities (Coronado et al., 2011; Kochanek et al., 2015; Rubiano et al., 2015). These numbers underestimate the impact as they do not include military casualties; the US Department of Defense reports 339,462 cases of TBI over the past 5 years (dvbic.dcoe.mil). TBI, also referred to as mechanical injury in this review, results in neuronal death often in the cerebrum, thereby producing several long-term and harmful symptoms including disturbances in memory, learning, attention, sleep, and motor coordination as well as the onset of headaches, fatigue, mood disorders, personality changes, and post-traumatic epilepsy (Rosenthal et al., 1998; Agrawal et al., 2006; Lowenstein, 2009; Hart et al., 2011).

For over a century, the writings of Cajal and other Nineteenth century neuroscientists instilled a dogma that the adult brain is incapable of replacing lost neurons. This belief was subverted when postnatal and adult stem cells in the mammalian subventricular zone (SVZ) and subgranular zone (SGZ) were rediscovered (Ihrie and Alvarez-Buylla, 2011; Spalding et al., 2013). While hippocampal SGZ neurogenesis is very interesting and especially relevant to the cognitive fluctuations in TBI, this review will focus on the SVZ as more information is known about its response to TBI and since SVZ progenitors migrate to injury sites (Young et al., 2011). The rodent SVZ replaces tens of thousands of olfactory bulb (OB) interneurons daily. Adult *Homo sapiens* and non-human primates also exhibit SVZ neurogenesis with similarities as well as significant differences from rodents (Eriksson et al., 1998; Bernier et al., 2000; Weickert et al., 2000; Kornack and Rakic, 2001b; Pencea et al., 2001b; Sanai et al., 2004; Curtis et al., 2007; Kam et al., 2009; Wang et al., 2011; Bergmann et al., 2012; Ernst et al., 2014). Nonetheless, the proximity of the SVZ to the cortex and other vital forebrain nuclei raises the possibility that the neurogenic potential of SVZ stem cells and their migratory progeny may naturally contribute to endogenous repair (Dizon and Szele, 2005; Young et al., 2011). Moreover, SVZ stem cells might be induced to respond more robustly.

Different strategies have been considered for harnessing the therapeutic potential of SVZ cells (Young et al., 2011). Endogenous SVZ cells might be induced via molecular manipulation *in situ* to proliferate, emigrate to sites of injury, and differentiate into the types of cells lost to TBI (Yu et al., 2013). SVZ cells might also be manipulated *in vitro* and then transplanted to the needed areas after they have been expanded and transduced with gene constructs to direct cell fate (Lois and Alvarez-Buylla, 1993; Kukekov et al., 1999; Ostenfeld et al., 2002; Gil-Perotin et al., 2013). It is important to consider how TBI alters the SVZ in the context of both strategies (Dizon and Szele, 2005).

A key question is if de novo mechanisms come into play after injury, if extant mechanisms are altered, or if some combination of the two occurs. It is unclear to what extent the same or different molecular regulators affect SVZ proliferation, migration, or survival in homeostasis as after TBI. In this review, we attempt to identify knowledge gaps and propose potential novel approaches. The SVZ field has exploded in the last decade (Dizon and Szele, 2005), and we have attempted to include most of the recent TBI-relevant citations. We acknowledge that many studies on the SVZ after stroke, neurodegenerative diseases, and other disorders may be relevant to TBI, but are too numerous to discuss here. By virtue of its plasticity, the postnatal brain may be a better target for repair than the adult. We concentrated on adults as there is less data on the young SVZ niche response to TBI, and as the SVZ changes markedly throughout postnatal life so comparisons across ages are problematic.

The SVZ responses to TBI can occur immediately or last for years and include mechanical forces (immediate), hemorrhage (short-term), edema (medium-term), and gliosis (medium to long-term). Each of these phases is accompanied by, or caused by, distinct molecular and cellular changes, suggesting that the SVZ is tightly regulated after TBI. These distinct temporal changes should inform therapeutic strategies and the selection of target molecules within the windows of opportunity.

Each individual TBI model is complex not just because of its temporal but because of its regional, molecular, and cellular variability. Inconsistency in the sorts of injuries and animals used in TBI studies lead to further difficulty in interpreting the results (Table 1). Injuries that extend to and include the SVZ or the rostral migratory stream (RMS) result in extensively different responses than parenchymal TBI (Ramaswamy et al., 2005). While cortical aspiration, weight percussion, and fluid percussion models of TBI do not directly lesion the SVZ (Szele and Chesselet, 1996; Holmin et al., 1997; Chirumamilla et al., 2002; Goings et al., 2002; Chen X. H. et al., 2003), the resultant mechanical shocks may affect the neurogenic niche, which should be taken into consideration. We have shown that different injury models (aspiration vs. thermocoagulatory cortical lesions) directed at the same brain region cause variable attempts at endogenous repair, which emphasized the necessity of studying the SVZ after different types of TBI and in multiple species (Szele and Chesselet, 1996; Goings et al., 2002, 2006; Ramaswamy et al., 2005; Sundholm-Peters et al., 2005).

**Table 1:** **Responses of the SVZ to mechanical brain injuries**.

**Type of injury**	**Species**	**Proliferation**	**Migration**	**Differentiation**	**References**
Knife cut through cerebral cortex, corpus callosum, and striatum	Rat	Increased number of cells, mitosis ND	ND	ND	Willis et al., 1976
Aspiration lesion of somatosensory cortex	Rat	Increased total number of cells, but no change in number of BrdU+ cells	No evidence of emigration to adjacent areas despite appearance of radial glia like fibers	Increased numbers of PSA-NCAM+ cells but no expression of mature markers in SVZ	Szele and Chesselet, 1996
Knife cut through cerebral cortex, corpus callosum, and fimbria fornix	Rat	Number of cells ND, increased LI	ND	Newborn cells in SVZ did not express markers of neurons, or glia	Weinstein et al., 1996
Weight percussion injury of cerebral cortex	Rat	ND	Emigration of nestin+/GFAP+ cells toward lesion in cortex	SVZ cells co-expressed nestin and GFAP	Holmin et al., 1997
Aspiration lesion of frontal cortex and olfactory peduncle	Mouse	Increased total number of cells and size of RMS, decreased number of BrdU+ cells	Rostral migration continued; emigration into anterior olfactory nucleus, frontal cortex	Increased number of calretinin+ cells indicating increased neuronal differentiation	Jankovski et al., 1998
Knife cut through RMS and cerebral cortex (separate rats)	Rat	Number of cells ND, increased number of BrdU+ cells	Rostral migration continued; emigration into cerebral cortex and striatum	PSA-NCAM+ cells in wound and striatum expressed GABA and TH	Alonso et al., 1999
Olfactory bulbectomy	Mouse	Number of cells ND, but increased size of RMS, decreased LI	Rostral migration continued, no emigration noted	ND	Kirschenbaum et al., 1999
Stab wound in cerebral cortex	Rat	Number of cells ND, non-significant transient increase in number of BrdU+ cells	ND	ND	Tzeng and Wu, 1999
Fluid percussion injury	Rat	Number of cells ND, increased number of BrdU+ cells	ND	Newborn cells in SVZ did not express markers of neurons, or glia	Chirumamilla et al., 2002
Aspiration lesion of somatosensory cortex	Mouse	Number of cells not changed, decreased number of BrdU+ cells	ND	ND	Goings et al., 2002
Fluid percussion injury	Rat	Number of cells ND, increased number of Ki67+ and PCNA+ cells at long survival times	ND	Increased numbers of neurofilament+ and GFAP+ cells 1 year after injury	Chen X. H. et al., 2003
Aspiration lesion of somatosensory cortex	Mouse	Number of cells ND, decreased number of retrovirally-labeled cells	Emigration into corpus callosum, and injured cortex	Differentiation into oligodendrocytes in corpus callosum, and astrocytes in lesioned cortex	Goings et al., 2004
Blunt steel needle stab	Mouse	ND	ND	Differentiation of SVZ neurospheres derived from both neonate and adult TBI brains promotes more GFAP rather than neurons and oligodendrocytes	Givogri et al., 2006
Aspiration lesion of somatosensory cortex	Mouse	No change in the number of BrdU+ cells in SVZ, but increased in CC	Rostral migration continued; emigration of newborn Dcx+ cells into corpus callosum and injured cortex	ND	Sundholm-Peters et al., 2005
Controlled cortical impact of cerebral cortex	Mouse	Number of cells ND, increased number of Ki67+ and BrdU+ cells on 1, 3, 7 DPI	ND	ND	Theus et al., 2010
Controlled cortical impact of cerebral cortex	Rat	Number of cells ND, increased number of Ki67+ cells	ND	Only trends of reduction for DCX+ cells in SVZ and SGZ	Acosta et al., 2013
Cortical contusion injury of cerebral cortex	Mouse	Number of cells ND, increased number of BrdU+ cells	Decreased number of BrdU+/NeuN+ cells in olfactory bulb; increased number of emigrating BrdU+ cells toward lesion in cortex but not NeuN positive	Newborn cells in SVZ did not express markers of neurons but glia (GFAP and IBA-1)	Radomski et al., 2013
Aspiration lesion of the motor cortex	Mouse	Increase in BrdU+ cells after 7 days, and decrease after 30 days	Emigration of Dcx+ cells into the injured cortex	SVZ derived cells differentiated in GFAP+, NeuN+ and Olig2+ cells in injured cortex	Saha et al., 2013
Traumatic axonal injury in corpus collosum	Mouse	Decreased total number of Gli1 positive cells in SVZ	ND	Number of NG2 progenitors increased in the cortex and corpus callosum; rarely colabeled with NG2 progenitors	Mierzwa et al., 2014
Cortical impact of cerebral cortex	Piglet	Number of cells ND, LI ND	Increased number of neuroblasts in the white matter of the hemisphere ipsilateral to the injury	No increase of total number of neuroblasts in the white matter	Costine et al., 2015

*Search key words: “SVZ” “Brain” “injury”—among 308 articles, the relevant studies are listed, ND, not determined*.

## Mechanical forces in traumatic brain injury

TBI involves physical forces that likely influence SVZ neurogenesis, usually including immediate but transient increases in intracranial compression and lateral ventricle (LV) pressure. Later, hemorrhage can cause obstructive hydrocephalus with persistently increased LV hydrostatic pressure. Human hydrocephalus can tear the ependymal layer and cause astrocytosis (Bruni et al., 1985; Sarnat, 1995). Parenchymal edema with or without obstructive hydrocephalus produces persistently increased intracranial pressure. Brain tissue loss, in contrast, can decrease pressure and increase LV volume (Volpe, 1995; Reider et al., 2002). These, and a long list of other macro and micro mechanical forces, are all important considerations as an emerging literature shows that mechanical stresses induce distinct molecular events (Ingber, 1997, 2002; Grodzinsky et al., 2000; Keller et al., 2003; Guirao et al., 2010).

Mechanical forces cause β-catenin homolog Armadillo to translocate to the fly nucleus (Farge, 2003). Wnt signaling affects SVZ neurogenesis and gliogenesis (Azim et al., 2014a,b), and β-catenin inhibits neuronal progenitor cell cycle exit and thus expands the cerebral cortex (Chenn and Walsh, 2002). Similarly, mouse embryonic stem cells subjected to sheer forces of different magnitude and duration exhibited concurrent changes in pluripotency and the Wnt signaling pathway (Wolfe et al., 2012). Mechanical forces are likely to affect not only SVZ cells but also ependymal cells (EC) as CSF hydrodynamic forces are necessary for their development (Guirao et al., 2010).

ECs form the barrier to the ventricles and are strongly linked by tight junctions (Mitro and Palkovits, 1981). Similarly, SVZ cells are connected by specific cell surface molecules (Lois et al., 1996) and are surrounded by a thin ECM and basal lamina distinct from other brain regions (Miragall et al., 1990; Jankovski and Sotelo, 1996; Mercier et al., 2002). Thus, the mechanical properties of ECs, the SVZ, and the adjacent tissue are all probably different from one another. It is unknown if SVZ or EC adhesion or ECM following TBI alters stiffness and thereby contributes to ventricular enlargement after TBI (Reider et al., 2002; Chen X. H. et al., 2003). However, ventricular expansion can be modeled, thereby making these questions tractable (Iwamoto et al., 1997; Bramlett and Dietrich, 2002). Rat kaolin-induced communicating hydrocephalus initially increased the number of SVZ nestin+ cells followed by a decrease (Li et al., 2014). The same model in ferret decreased SVZ Ki67+ cells in SVZ and increased SVZ caspase-3+ cells (apoptosis) (Di Curzio et al., 2013). Thus, future studies should be able to integrate post-TBI mechanical forces with molecular events.

## SVZ proliferation and neurogenesis generally increase after traumatic brain injury

**Figure 1:**
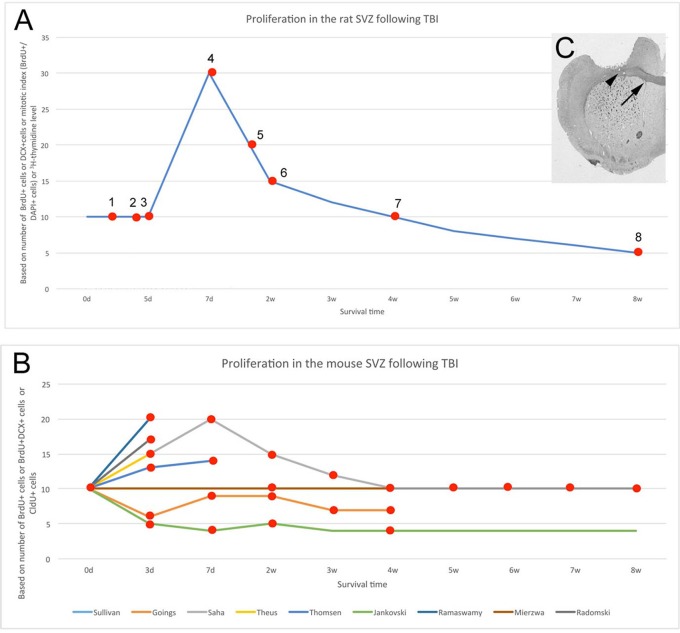
**Altered SVZ proliferation is variable across species and experiments. (A)** Increased SVZ proliferation and/or neurogenesis in rat after mechanical injury to brain. Data from multiple studies. 1 - (Tzeng and Wu, 1999), 2 - (Goodus et al., 2015), 3 - (Gotts and Chesselet, 2005), 4 - (Szele and Chesselet, 1996; Sun et al., 2009, 2010; Bye et al., 2011), 5 - (Weinstein et al., 1996) 6, 7 - (Bye et al., 2011; Grande et al., 2013), 8 - (Acosta et al., 2013, 2014). For both **(A,B)**, baseline was set at a value of 10 and percent changes from this are shown. **(B)** SVZ proliferation or neurogenesis in mouse after mechanical injury across multiple studies. Individual studies shown with colored lines (Jankovski et al., 1998; Goings et al., 2002; Ramaswamy et al., 2005; Theus et al., 2010; Radomski et al., 2013; Saha et al., 2013; Sullivan et al., 2013; Mierzwa et al., 2014; Thomsen et al., 2014). The Saha et al., study was the most complete including time points at 3 days, 1, 2, 3, 4, 5, 6, 7, and 8 weeks post-lesion. **(C)**. Cortical injury via aspiration. The injury excludes the corpus callosum (arrowhead) but is close to the subventricular zone (arrow). Aspiration lesions of the cerebral cortex were made in the same location both in mouse and rat (Szele and Chesselet, 1996; Goings et al., 2002, 2004).

Changes in cell numbers and proliferation in the SVZ after knife cut brain injury were already investigated in the mid-1970s in rat (Willis et al., 1976) and have been studied more often than other sequelae of TBI (Table 1). 5-Bromo-2′-deoxyuridine (BrdU) pulse studies at various time points after injury indicate the number of cells in S-phase and assume that the cell cycle will be completed and mitosis will occur. It is important to determine the percent of proliferating SVZ cells, or the “labeling index,” as the final population of SVZ cells is ultimately determined by the combination of the labeling index, cell cycle length, emigration rates from the SVZ, and cell death. That is, more BrdU+ cells do not necessarily mean increased SVZ cell numbers. With these caveats in mind, a survey of the literature suggests that SVZ division and cell numbers increase after TBI although there are discrepancies amongst studies (Figure 1).

The following parameters are amongst the most crucial to consider in comparing different post-TBI SVZ proliferation studies: (1) species, (2) type of injury, (3) proliferative markers, and (4) survival time. Contradictory results can originate from the diversity of these parameters. Considering the survival time, a four-phase response to TBI may help explain apparent discrepancies across studies (Figure 1A). Within the first 5 days following TBI, SVZ proliferation in the adult rat is unchanged (Tzeng and Wu, 1999; Gotts and Chesselet, 2005; Goodus et al., 2015), thereafter proliferation is increased, peaking around the post-operative 7th day with a 100% increase (Szele and Chesselet, 1996; Weinstein et al., 1996; Gotts and Chesselet, 2005; Sun et al., 2009, 2010; Bye et al., 2011) and subsequently returns to baseline by the end of the first month (Bye et al., 2011). More recently, the timepoint of 7 and 14 days after aspiration cortical lesions in rat was studied. A significant increase in BrdU+ cells ipsilateral compared to contralateral to the lesion was observed at 7 days, but this difference was lost by 14 days (Yang et al., 2010). Chronically, (8 weeks and on) baseline proliferation is further decreased by ~50% after controlled cortical impact in rats (Acosta et al., 2013, 2014). These phenomena (Figure 1A) highlight the importance of careful interpretation of results that are often obtained from different survival periods.

In contrast to rat, a four-phase process cannot explain the discrepancies in mouse studies of SVZ proliferation post-TBI (Figure 1B). Most articles report increased SVZ proliferation following controlled cortical impact or aspiration lesion (Theus et al., 2010; Radomski et al., 2013; Saha et al., 2013; Thomsen et al., 2014). However, no change was found by the Armstrong group (Sullivan et al., 2013; Mierzwa et al., 2014), and the Szele group found a biphasic decrease that occurred in mouse in the first post-operative week and later in the fourth and fifth post-operative weeks (Goings et al., 2002). This decrease is similar to the observations of Jankovski and colleagues in mouse after lesion of the cortex and olfactory peduncle (Jankovski et al., 1998). The reason for diverse responses of SVZ proliferation after injury was further investigated, and Radomski and colleagues clearly demonstrated in mouse that SVZ proliferation did not change after 3 days of a “mild” cortical impact (up to 1 mm depth) but “moderate” impact (up to 2 mm depth) caused a significant 60% increase (Radomski et al., 2013). This example suggested that severity of injury affects the SVZ proliferation instead of injury timing (Figure 1B).

The extensively used fluid percussion model of TBI involves a controlled impact on the cerebral cortex surface (McIntosh et al., 1987; Chen S. et al., 2003; Chen X. H. et al., 2003; Prins and Hovda, 2003). This TBI model has several strengths such as the control of its size and location and is considered to be one of the best of non-penetrating TBI models. SVZ cell division was augmented at short time points after fluid percussion injury in rat (Chirumamilla et al., 2002). Another group found that control rats had significant age-related declines in PCNA+ and Ki67+ SVZ cell numbers, but this decline was blocked in fluid percussion-injured rats (Chen X. H. et al., 2003). This model is mostly been used in rat but has also been adapted to mice (Carbonell et al., 1998). We found more murine SVZ cells in S phase after controlled cortical impact of the cortex (Ramaswamy et al., 2005). Penetrating brain injury models that do not impact the SVZ are similar to certain human brain injuries as well to brain surgeries/biopsies and thus deserve to be studied. Sagittal cuts through the cortex and fimbria doubled the number of cycling SVZ cells the rat SVZ (Weinstein et al., 1996). Stab wounds of the rat cerebral cortex did not alter BrdU-incorporation in the SVZ (Tzeng and Wu, 1999); however, a similar needle stab in mouse induced a 2-fold increase in SVZ BrdU+ cells (Givogri et al., 2006). Thus, in comparison to more dramatic lesions, smaller injuries affect SVZ proliferation much less or not at all.

Considerable data have accumulated showing constitutive human SVZ proliferation. SVZ neurogenesis in the first 2 years after birth is widely accepted, but neurogenesis in the adult human SVZ is controversial. According to Sanai and colleagues, adult human neurogenesis is largely depleted along the SVZ–RMS–olfactory bulb axis after 18 months (Sanai et al., 2011). Nevertheless, it still seems to exist in at least a vestigial form, as Wang and colleagues found proliferation in the ventral SVZ and a small number of migrating DCX+ cells in the RMS and olfactory tract in adult humans (Wang et al., 2011). In contrast, Frisen and colleagues did not find evidence for olfactory bulb neurogenesis but instead found evidence for proliferative SVZ cells as well as adult striatal neurogenesis by birth dating of neurons based on the detection of ^14^C levels in the brain (Bergmann et al., 2012; Ernst et al., 2014). This creative technique may prove to be important in determining the human neurogenic response to TBI. Indeed, the same group showed that in Huntington's disease, the number of newborn neurons appeared to decrease (Ernst et al., 2014). It will be important to confirm or refute the ^14^C studies to help understand the capacity of human neurogenesis to contribute to TBI brain repair.

Relatively few investigations have evaluated human SVZ proliferation following TBI. There was no increase in the number of DCX+ migrating neuroblasts in the SVZ of children with TBI compared to non-TBI patients (Taylor et al., 2013). However, caution is warranted due to the small sample size (6 TBI vs. 7 non-TBI) and the short survival time (average 34 h) examined. The one patient who survived 7 days in this study had the highest number of migrating neuroblasts, thereby indicating a need for future studies of longer term responses to TBI. Adult human patients following TBI exhibited increased numbers of DCX+, PSA-NCAM+, and SOX2+ cells (Zheng et al., 2013). Nonetheless, this study focused on the cortex and not the SVZ. Taken the aforementioned data together, a shift from rodent models to primate research and more post-mortem human studies are needed in order to understand the proliferative capacity of the human SVZ and to harness this potentially relevant endogenous resource for the repair of injured brain.

Despite over 100 therapies attempting to ameliorate TBI in rodents, none has been successfully translated to the clinic (Marklund et al., 2006; Margulies et al., 2009). This could be due to interspecies differences such as lissencephalic vs. gyrencephalic cortices: growth trajectory, cellular proliferation, and myelination (Costine et al., 2015). In searching for a better human SVZ model, neurogenic properties of other species have been described: rabbit (Luzzati et al., 2006), sheep (Liard et al., 2009; Brus et al., 2012; Low et al., 2013), pig (Liard et al., 2009), and non-human primate (Kornack and Rakic, 2001a; Pencea et al., 2001b; Bedard et al., 2002; Gil-Perotin et al., 2009). However, these species have not been systematically investigated and their SVZ repair capacity is still unknown (with the exception of increased proliferation found in the immature piglet SVZ) (Costine et al., 2015).

## Progenitor cells migrate from the subventricular zone to traumatic brain injury

**Figure 2:**
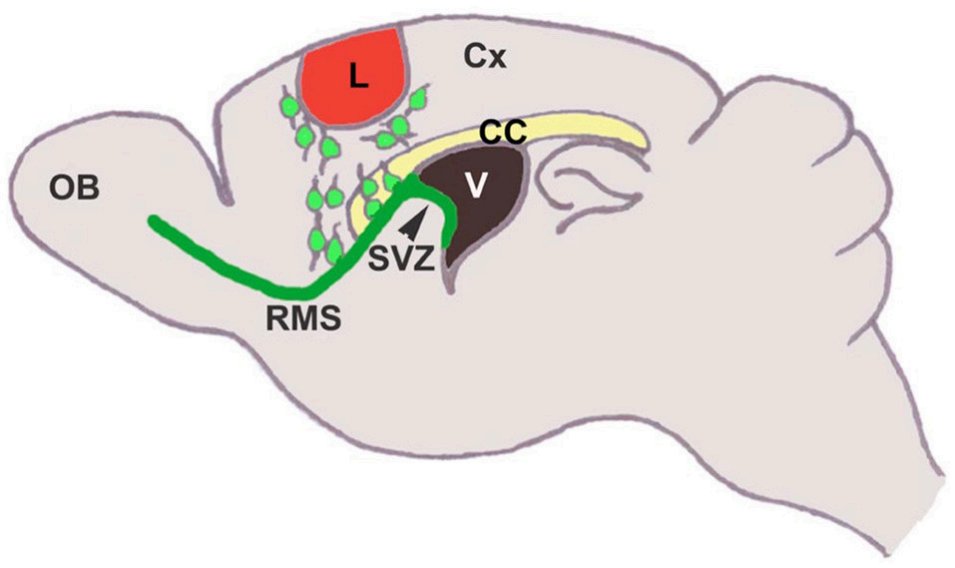
**SVZ neural stem cells differentiate into neuroblasts, that migrate tangentially through the rostral migratory stream to the olfactory bulb, where they migrate radially and differentiate into interneurons**. In response to injury, these cells migrate from the SVZ/RMS to the injury site in an attempt to regenerate the damaged tissue (Saha et al., 2012).

For stem cells to be of therapeutic value, they must move to or be placed into areas of injury. SVZ cells migrate to brain injuries to variable degrees, and this migration is regulated via several mechanisms (Young et al., 2011). Neuroblasts in the adult SVZ normally migrate to the OB in strings, which is termed as chain migration in the confined RMS. The RMS contains a network of specialized astrocytes (Jankovski and Sotelo, 1996; Lois et al., 1996) that guide neuroblast migration but are not absolutely necessary for their movement (Wichterle et al., 1997; Jacques et al., 1998). TBI induces both chain and individual cell migration out of the SVZ and RMS (Figure 2).

**Figure 3:**
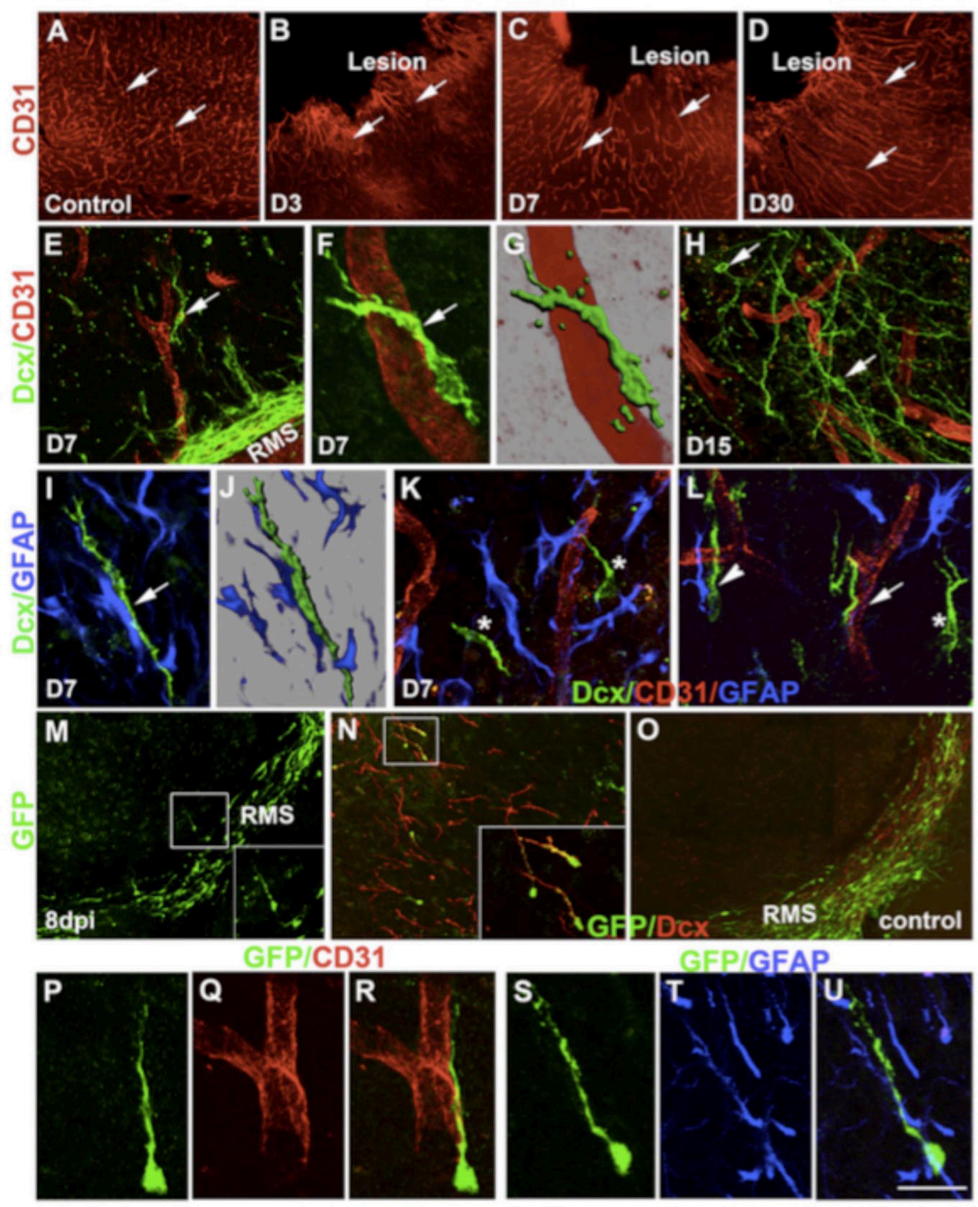
**Changes in cortical microenvironment assist progenitor migration after lesion**. CD31 immunostaining of control intact cortex **(A)** and lesioned cortex at different times after injury **(B–D)**. Double immunofluorescence confocal micrograph of Dcx (green) and CD31 (red) at day 7 **(E–G)** and day 15 **(H)**. Similarly, double-immunostaining of astrocytes (blue) and neural progenitors (green) after 7 days of lesion **(I,J)**. (**K**,**L** asterisk) Migration of progenitors (green) without any association. GFP immunostaining of brain sections 8 days after lentivirus injection (dpi) into the SVZ/RMS **(M)**. In the cortex, GFP+ cells (green) co-express Dcx (red) (boxed in **N**). GFP+ cells remain within the RMS in control brains **(O)**. GFP+ progenitors also showed association with either blood vessels **(P–R)** or astrocytes **(S–U)**. Scale bars: **(A–D)** 250 μm, **(E,H)** 50 μm, **(F,G)** 16 μm, **(I,J,P–U)** 20 μm, **(K,L)** 30 μm, **(N)** 80 μm, (**N** inset) 40 μm, **(M,O)** 100 μm, (**M** inset) 50 μm (Saha et al., 2013).

Classic studies showed that RMS transection causes SVZ cell accumulation posterior to the lesion and cell emigration into the lesioned cortex and striatum in mouse (Jankovski et al., 1998; Kirschenbaum et al., 1999) and rat (Alonso et al., 1999). When the PSA-NCAM+ SVZ neuroblasts migrated to the lesion, they remained in their normal chain-like configuration (Lois et al., 1996). Removal of the OB resulted in continued rostral migration of SVZ cells (Jankovski et al., 1998; Kirschenbaum et al., 1999), although rostral migration is induced by OB chemoattractants (Liu and Rao, 2003; Ng et al., 2005). In a motor cortex aspiration TBI model in mouse (Figure 3), neuroblasts continued to migrate in chains after exiting the RMS and entering the corpus callosum, but as soon as they reached the cortex, they no longer migrated as chains (Saha et al., 2013). Most were found in close proximity or in contact with blood vessels (Figures 3E–H) and GFAP+ reactive astrocytes (Figures 3I–L), although a portion of neuroblasts was not associated with either of these cell types (Figures 3M–U), suggesting that multiple modes of migration may be present after injury (Saha et al., 2013).

Do human SVZ cells emigrate after injury? DCX+ cells in the SVZ decreased as patient age increased in children after TBI (Taylor et al., 2013). The density of immature migrating neuroblasts in infants (under 1 year of age) was significantly greater than in young (2–6 years of age) and older (7–10 years of age) children (Taylor et al., 2013). The DCX+ cells had migratory morphology and did not co-localize with markers for astrocytes, microglia, or macrophages in the periventricular white matter.

Quite a number of studies in the past decades suggest that SVZ cell emigration to TBI is guided by diffusible proteins (chemoattractants) secreted by cells in the injured region. In response to unilateral aspiration cortical lesion, Saha and colleagues demonstrated that SVZ neuroblast emigration toward injury is due to reactive astrocyte-derived SDF-1 signaling (Saha et al., 2013). SDF-1 is a key regulator of directed migration after injury as well as the homing of neural stem cells and increased mobility of neuroblasts (Kokovay et al., 2010; Moon et al., 2013). In a mouse model of TBI coupling stab wound and cryogenic injury, an SDF-1 analog increased the number of neuroblasts reaching the injury (Filippo et al., 2013). Moreover, the presence of analog increased SDF-1 expression, suggesting a positive feedback loop that maintains a chemoattractant gradient (Filippo et al., 2013). Similarly, the blockade of SDF-1/CXCR4 signaling after TBI using the CXCR4 antagonist AMD3100 led to fewer neuroblasts reaching the injury site. Finally, SDF-1 was also shown to increase phosphorylation of ERM (ezrin-radixin-moesin) proteins in migrating neuroblasts in response to TBI, helping to maintain the migratory morphology of these cells (Moon et al., 2013). Clearly, exogenous modulation of chemokine signaling may evolve into a key way to mobilize endogenous SVZ-mediated repair.

Most experiments studying SVZ cell migration after brain injury have utilized fluorescent cell labeling or tagged cells injected directly into the lateral ventricle or the SVZ. A library of retroviral vectors developed in the Cepko laboratory that was originally used in the SVZ to examine migration patterns and lineage relationships after cortical injury (Goings et al., 2004) has been recently used to show the early embryonic origin of SVZ stem cells (Fuentealba et al., 2015). Goings and colleagues showed that a portion of SVZ cells migrated toward the injury 4 days after aspiration cortical lesions and were still detected at 3 weeks after injury (Goings et al., 2004).

## Traumatic brain injury effects on SVZ cell survival and differentiation

A growing body of evidence suggests that cells may survive better in the SVZ after TBI and mature for weeks after emigration to TBI (Table 1). The number of TUNEL+ SVZ cells was reduced 3 days post-controlled cortical impact injury in ephrinB3^−∕−^ and EphB3^−∕−^ mice (Theus et al., 2010). SVZ cell survival was also increased by administration of basic fibroblast growth factor (FGF2) but not epidermal growth factor (EGF) for 7 days following brain injury (Sun et al., 2009, 2010). FGF2 administration after fluid percussion injury in mouse increased newborn cell survival up to 4 weeks post injury, and the majority of survival was of neurons (Sun et al., 2009).

For regenerative value, not only do SVZ cells have to survive but they also have to differentiate appropriately after TBI. A recent study using tamoxifen-inducible Gli1-CreERT2;R26-YFP bi-transgenic mice demonstrated that the number of newborn SVZ cells increased post-controlled cortical impact brain injury, but few were found in the lesion area (Mierzwa et al., 2014). Six weeks post TBI, the number of YFP+/Dcx+ double positive cells was still not significantly changed, yet there was a slight increase in the number of Dcx+ cells observed in the dorsolateral SVZ (Mierzwa et al., 2014). In another study, neuroblasts migrating from the SVZ into the neocortex 2 weeks after cortical injury became proliferative (BrdU+/Dcx+), but very few new mature neurons were found adjacent to the lesion 28 days after the injury (Goodus et al., 2015). In piglets, injured at postnatal day 7, cortical impact did not increase the total number of neuroblasts in white matter. However, 14 days post–injury, the number of Dcx+ cells increased (Costine et al., 2015). Taken together, the data suggested that the stem cell niche (extrinsic factors) limits SVZ differentiation both before and after mechanical brain injuries.

Once the SVZ cells migrate out of the niche environment, they could also differentiate into astrocytes and oligodendrocytes, which are known to migrate into the forebrain in late brain development (Levison and Goldman, 1993). The retroviral lineage studies described above showed that SVZ cells that migrated to the corpus callosum after aspiration cortical lesions assumed oligodendrocytic morphology, whereas those that had moved near the cortical lesion became astrocytes (Goings et al., 2004). The predominant glial differentiation of SVZ cells also occurs in adult mouse brain after cortical contusion injury. BrdU+ cells homing to the ipsilateral cortex became either IBA-1+ or GFAP+ cells, but not NeuN+ cells (Radomski et al., 2013). These data indicate that TBI induces progenitors to move out of the SVZ and become glia in rat (Chirumamilla et al., 2002) and mouse (Goings et al., 2004), although most reactive astrocytes in response to TBI are generated locally.

## Molecular regulation of SVZ cells after traumatic brain injury

Since there does not seem to be significant SVZ-derived neuronal replacement after mechanical injuries, various molecular and cellular treatments have attempted to boost neurogenesis. Single or multiple doses of activated protein C injected intraperitoneally in mice promoted post-controlled cortical impact injury proliferation of neuroblasts in the SVZ and migration to the perilesional area (Petraglia et al., 2010). Granulocyte colony-stimulating factor (GCSF) delivered via osmotic pumps after fluid percussion injury in rats resulted in significantly increased Dcx+ cell numbers in the ipsilateral SVZ (Yang et al., 2010). In another study, GCSF and/or human umbilical cord blood cell (hUBC) therapy was administered 1 week after controlled cortical impact (Acosta et al., 2014). Eight weeks later, the number of SVZ and hippocampal Dcx+ cells was significantly increased in all cohorts—hUCB, GCSF, and hUCB/GCSF. The therapeutic effect was also demonstrated via reduced hippocampal cell loss (Acosta et al., 2014). It is likely that during the next decade, significant improvements will be attained in exogenous stimulation of the SVZ's natural repair processes (Ihrie and Alvarez-Buylla, 2011).

A number of classic studies examined the effects of exogenous growth factors on SVZ neurogenesis, and they typically increased SVZ cell numbers in adjacent structures, suggesting induced emigration (Craig et al., 1996; Kuhn et al., 1997; Benraiss et al., 2001; Pencea et al., 2001a). BDNF or BDNF small molecule mimics caused SVZ cells to emigrate toward nearby nuclei (Benraiss et al., 2001; Pencea et al., 2001a; Fon et al., 2014) in a concentration-dependent manner (Petridis and El Maarouf, 2011). EGF and FGF2 injected into the lateral ventricles increased SVZ progenitor proliferation (Craig et al., 1996; Kuhn et al., 1997). EGF also caused SVZ cell emigration to the cerebral cortex, striatum, corpus callosum, and septum, and subsequent differentiation into astrocytes and oligodendrocytes (Craig et al., 1996; Gonzalez-Perez et al., 2009). In Craig et al. study, TGF-α caused SVZ cell emigration into the striatum (Craig et al., 1996). Appearance of newborn cells could theoretically result from activation of latent neurogenic programs outside the SVZ (Palmer et al., 1999; Buffo et al., 2008; Amamoto and Arlotta, 2014), but it is unlikely that injection of growth factors alone could induce neurogenesis in the parenchyma (Grande et al., 2013).

In addition to the diffusible molecules described above, ECM and cell surface adhesion molecules regulate neural development. The SVZ expresses such molecules even after the rest of the brain has lost their expression postnatally (Miragall et al., 1990; Szele et al., 1994; Gates et al., 1995; Thomas et al., 1996). Many ECM molecules are made by astrocytes and regulate SVZ neuroblast motility (Husmann et al., 1992; Martoncikova et al., 2014; Giblin and Midwood, 2015). The ECM is enzymatically processed by matrix metalloproteinases (MMP) that are essential in regulating SVZ NSC quiescence, and identity and inhibition of MMPs limit neuroblast emigration from the SVZ to the striatum in stroke (Lee et al., 2006; Kang et al., 2008; Porlan et al., 2014). Although not an adhesion or ECM molecule, the cell transmembrane proteins EphrinB3-EphB3 control SVZ NSC proliferation via p53 in TBI in mouse (Theus et al., 2010). Taken together, the data suggest that regulation of the ECM and adhesive cell surface molecules in the SVZ is complicated, but important after TBI.

SVZ neural stem/progenitor cells are also regulated by intracellular transcriptional and epigenetic factors (Ihrie and Alvarez-Buylla, 2011). The transcription factor Gata3, for instance, can be induced in radial glial (NSC) of adult Zebrafish by stab wound lesions. Gata3 expression is FGF-dependent and is necessary for the reactive proliferation of radial glia and migration of newborn neurons in the regenerating telencephalon (Kizil et al., 2012). However, no evidence has shown the upregulation of Gata3 in the rodent SVZ after TBI (Yoshiya et al., 2003). In neuroblastoma, Gata3 is reported to activate Cyclin D1 and promote proliferation, whereas silencing of Gata3 induces differentiation (Molenaar et al., 2010; Peng et al., 2015). It could be worthwhile to examine whether exogenous activation of Gata3 in the rodent SVZ enhances the neurogenic ability of neural stem cells after mechanical injury. Compared to post-injury responses, far more transcriptional mechanisms have been reported in regulation of constitutive postnatal and adult SVZ neurogenesis (Hsieh, 2012; Urban and Guillemot, 2014).

It seems that exogenous manipulation of transcription factors may become crucial for brain repair, thereby considering the restriction of neuronal subtypes generated by neuroblasts that have migrated out of the SVZ/RMS/OB system (Teramoto et al., 2003; Liu et al., 2009; Young et al., 2011). For example, Fezf2 is required for layer 5 subcortical projection neuron development, and overexpression of Fezf2 in the postnatal SVZ is able to reprogram newborn cells from GABAergic interneurons into glutamatergic projection neurons (Kwan et al., 2012; Zuccotti et al., 2014). Moreover, recent studies have suggested that reactive astrocytes have stem cell potential after invasive brain injury (Buffo et al., 2008; Robel et al., 2011). Functional inactivation of Olig2 or overexpression of either Sox2 or NeuroD1 in reactive astrocytes has successfully generated neurons *in vivo* (Buffo et al., 2005; Guo et al., 2013; Niu et al., 2013; Su et al., 2014). A systematic analysis of the roles of multiple transcription factors will facilitate the development of new brain repair therapies including *in situ* direct conversion of astrocytes (Arlotta et al., 2003; Amamoto and Arlotta, 2014).

In addition to transcription factors, a number of epigenetic regulators have been highlighted during SVZ neurogenesis (Hsieh, 2012; Urban and Guillemot, 2014). Chromatin modifiers, such as Ezh2, Jmjd3, Mll1, CHD7, and the BAF complex, contribute to proper neurogenesis and differentiation in the SVZ/RMS/OB (Lim et al., 2009; Feng et al., 2013; Ninkovic et al., 2013; Hwang et al., 2014; Park et al., 2014). Nevertheless, few of them have been studied in the SVZ response to mechanical brain injury. From the clinical perspective, activators and inhibitors of different epigenetic modification enzymes are commercially available. For example, the histone deacetylase inhibitor, sodium butyrate, is reported to elevate SVZ cell proliferation and enhance neurogenesis after stroke (Kim et al., 2009).

Grafting cells into cerebral cortex lesion cavities has indicated that cells can survive and develop new connections. Several papers have demonstrated that developing cells can be grafted into rodent TBIs (Castro et al., 1987, 1988; Girman and Golovina, 1988; Kolb et al., 1988; Sorensen et al., 1990). Engraftment of pluripotent stem cell-derived neural cells or even mesenchymal stem cells or bone marrow mononuclear cells into cortical lesions has also been studied in rodents (Gaillard et al., 2007; de Freitas et al., 2012; Espuny-Camacho et al., 2013; Tajiri et al., 2013; de Freitas et al., 2015; Michelsen et al., 2015). Overall these remarkable studies show that engrafted cells can survive, project axons into appropriate regions, and participate in at least some functional recovery. They also suggest that SVZ cells could be induced to perform similar feats of repair.

## Conclusion

The number of laboratories studying the potential regenerative abilities of rodent and human SVZ for cell replacement in mechanical injuries has significantly expanded in the last two decades. Most studies of lesions adjacent to or including the SVZ resulted in the alteration of SVZ biology with some combination of cell proliferation, migration, survival, or differentiation. These changes and differences can be explained by the level of severity, location, timing, or types of injury. Several different research groups using well-characterized injury models found similar or opposite features of SVZ cell property. Thus, generalization of SVZ response should be avoided, especially when comparing studies done in different species. To better understand the dynamics of SVZ cell plasticity, several researchers have analyzed intra- and extracellular mechanisms. Besides the well-recognized growth factors in SVZ biology including EGF and FGF2, TGF-alpha and MMPs are involved in neuroblast emigration. In addition, the roles of several transcription factors (Gata3, Fezf2, Olig2) and epigenetic regulators (Ezh2, Jmjd3, Mll1, CHD7, BAF) in the SVZ govern SVZ cell proliferation and differentiation. Taken together, the field is now poised to translate some of the fundamental findings into clinically relevant treatment modalities designed to augment SVZ neurogenesis, emigration, and neural repair.

## Author contributions

EC wrote review, composed figures and table. IA wrote review, composed figures. MM wrote review, composed figures. BS wrote review. MD wrote review. FS wrote review.

## Funding

MD supported by NIH RO1 NIH/NINDS R01NS086945-01. FS supported by MRC/NNSFC project grant MR/M010554/1. BS supported by China Scholarship Council and Henry Lester Trust. MM support by FAPESP 2012/06810-1 and 2014/00927-0.

### Conflict of interest statement

The authors declare that the research was conducted in the absence of any commercial or financial relationships that could be construed as a potential conflict of interest.
